# A positive feedback loop between KPNA2 and FOXM1 promotes the proliferation of lung adenocarcinoma

**DOI:** 10.1186/s40001-025-03674-1

**Published:** 2025-12-18

**Authors:** Ying Wang, Yan Jin, Xin Li

**Affiliations:** 1 Respiratory Department, The First Affiliated Hospital of Jinzhou Medical University, Jinzhou, 121000 Liaoning China; 2 Respiratory Department, Nantong Sixth People’s Hospital, No. 881, Yonghe Road, Nantong, Jiangsu China

**Keywords:** Lung adenocarcinoma, KPNA2, Proliferation, FOXM1, Positive feedback loop

## Abstract

Lung adenocarcinoma is a prevalent form of non-small cell lung cancer (NSCLC) that accounts for approximately 40% of all lung cancers. However, despite significant therapeutic advances in recent years, the overall survival of patients with lung adenocarcinoma remains poor. Consequently, it is necessary to identify further novel targets and drugs that can contribute to enhancing the prognosis of these patients. To this end, in this study, using siRNA silencing, we initially knocked down KPNA2 in lung adenocarcinoma cell lines, and performed CCK8 and 5-ethynyl-2ʹ-deoxyuridine assays to examine the proliferation of lung adenocarcinoma. Chromatin immunoprecipitation was used to determine whether the transcription factor FOXM1 can bind to the promoter region of KPNA2, and we used AlphaFold3 to predict the proteins that bind to KPNA2 and to characterize the binding sites on these proteins. We found that KPNA2 is upregulated in lung adenocarcinoma and that inhibiting its expression effectively reduced the proliferation of lung adenocarcinoma cancer cells. Both bioinformatics analyses and experimental findings provided evidence that the expression of KPNA2 is directly controlled by FOXM1. Furthermore, we observed a reduction in FOXM1 expression following the suppression of KPNA2. These findings indicate the presence of a positive feedback loop between the expression of KPNA2 and FOXM1, which contributes to an enhanced proliferation of lung adenocarcinoma cells.

**Supplementary Information:**

The online version contains supplementary material available at 10.1186/s40001-025-03674-1.

## Introduction

Lung adenocarcinoma is a prevalent form of non-small cell lung cancer (NSCLC) that accounts for approximately 40% of all lung cancer diagnoses in North America [1]. This type of cancer originates from the alveolar cells within the small air sacs of the lungs [2]. It can arise in the smaller bronchi or airways and may also expand beyond the trachea or spread across the alveolar walls. The causes of lung adenocarcinoma are complex and may involve diverse contributary factors, including smoking, occupational exposure, air pollution, heredity, and a history of chronic lung diseases [3]. Treatment approaches for lung adenocarcinoma include surgical removal, radiation therapy, chemotherapy, targeted therapy, and immunotherapy [4]. At the molecular level, gene mutations, such as that of KRAS, are considered to be an important origin of these tumors [5]. Accordingly, a better understanding of the regulatory mechanisms of lung adenocarcinoma is importance for the treatment of this disease.

Among the different genetic drivers of lung adenocarcinoma, the nuclear transport protein karyopherin subunit alpha 2 (KPNA2) has emerged as a potential oncogene**.** The *KPNA2* gene, also referred to as importin alpha 114, is a member of the karyopherin family, the members of which play roles in cytoplasmic–nuclear transport. KPNA2 is also implicated in a range of other cellular processes, including cell differentiation, proliferation, apoptosis, transcriptional regulation, immune response, and viral infection [6], and has also been established to play roles in the subcellular localization of key DNA damage response (DDR) proteins, with the expression of these proteins in the cytoplasm being associated with that of KPNA2 [7]. Notably, it has been shown that the expression of KPNA2 is upregulated in different types of malignant tumors, and its abnormal expression is generally associated with a poor prognosis in patients, indicating that KPNA2 may play an important role in oncogenesis and tumor progression [8, 9]. Accordingly, the abnormally high levels of KPNA2 during tumorigenesis and its potential role as a biomarker identify this protein as a potential target for cancer treatment.

In this study, we examined the function and regulatory mechanisms of KPNA2 in lung adenocarcinoma. Our findings revealed that KPNA2 is upregulated during cancer development and that inhibiting its expression effectively reduced the proliferation of cancer cells. Both the results of bioinformatics analysis and experimental findings indicated that expression of KPNA2 is directly controlled by FOXM1. Furthermore, we observed a reduction in expression of FOXM1 following a suppression of KPNA2. These findings thus provide evidence to indicate the operation of a positive feedback loop via which the expression of KPNA2 and FOXM1 is mutually regulated, and may contribute to enhancing the proliferation of lung adenocarcinoma cells.

## Materials and methods

### Cell culture

Lung adenocarcinoma cell lines (A549 and H1299) were purchased from the Shanghai Cell Bank, Chinese Academy of Science. Cells were characterized and authenticated by STR. All the cells were cultured in Dulbecco’s modified Eagle’s medium (11,965,092; Gibco, USA) containing 10% fetal bovine serum (A5669701; Gibco), and maintained a humidified incubator at 37 °C in a 5% CO_2_ atmosphere.

### siRNA transduction

Small interfering RNAs (siRNAs) KPNA2-1 (5′-AAUCUUACCUGGACACUUU-3′) and KPNA2-2 (5′-UUCGUUAAGCUUAAUUGAGAA-3′) [10], FOXM1 (#1, stB0001139A-1–5; #2, stB0001139B-1–5) and control siRNA (siN0000002-1–5) were purchased from ribobio Co., Ltd (Guangzhou, China), and were used to transfect cells using a riboFECT CP Transfection Kit (ribobio).

### CCK8 assay

The A549 and H1299 cell lines were seeded at a density of 6000 cells per well in 96-well cell culture plates, and subjected to different treatments. Following incubation for 0, 24, 48, 72, 96, and 120 h, the culture supernatants were carefully removed and mixed with CCK8 reagent (Dojindo, Kumamoto, Japan). Subsequently, the absorbance was measured at 450 nm following the manufacturer’s instructions.

### Western blot analysis

Cells were lysed using RIPA lysis buffer to release proteins. The resulting lysates were centrifuged, and the supernatants thus obtained were collected. Samples of these supernatants were added to 5 × sample buffer and boiled for 10 min. The proteins were separated on SDS–PAGE gels and thereafter transferred onto PVDF membranes. These membranes were subsequently incubated overnight with specific primary antibodies (KPNA2, #14,372, CST; FOXM1, #20,459, CST; ACTIN, #8457, and CST) at 4 °C, and thereafter incubated with the corresponding secondary antibodies at room temperature for 1 h. Appropriate imaging equipment was used to visualize the target protein bands.

### RT–PCR

RNA was extracted from cells using TRIZOL reagent and was subsequently reverse transcribed and subjected to RT–PCR analysis using dedicated kits (TaKaRa). The primers were designed using a web resource (http://pga.mgh.harvard.edu/primerbank/) [11], the sequences pf which are as follows: KPNA2-F: 5′-CTGCCCGTCTTCACAGATTCA-3′, KPNA2-R 5′-GCGGAGAAGTAGCATCATCAGG-3′, FOXM1-F: 5′-GGAGCAGCGACAGGTTAAGG-3′, FOXM1-R: 5′-GTTGATGGCGAATTGTATCATGG-3′, ACTIN-F: 5′-CATGTACGTTGCTATCCAGGC-3′, ACTIN-R: 5′-CTCCTTAATGTCACGCACGAT-3′.

### Chromatin immunoprecipitation

Cells (2 × 10^7^) were processed using a ChIP kit (9003 s; CST) according to the manufacturer’s instructions, with the supernatants being collected and incubated overnight at 4 °C with anti-H3, IgG, or anti-FOXM1 (#20,459; CST) antibodies. The primers used for detection were as follows: KPNA2-CHIP-F: 5′-ACGGTCTTTGAGCTGAGTCG-3′, KPNA2-CHIP-R: 5′-GAGTCTGTACCTGCGAAGCG-3′.

### EDU proliferation assay

Cells subjected to different treatments were plated in 6-well plates and treated with EDU for 4 h, after which they were processed according to the manual of iClick EdU Andy Fluor 647 (GeneCopoeia, USA).

### Expression and survival analyses

TCGA expression data were downloaded from UCSC Xena (https://xenabrowser.net/heatmap/#), and survival analysis was downloaded from UCSC Xena (https://xenabrowser.net/heatmap/#) and the website http://kmplot.com/analysis/index.php.

### Protein docking

Analyses of the interactions between KPNA2 and CHEK2, and CHEK2 and FOXM1 were performed using AlphaFold3 [12], and three-dimensional structural diagrams were generated with reference to the following website: https://www.rcsb.org/3d-view/.

### Co-immunoprecipitation (Co-IP)

A549 cells lysates were prepared using lysis buffer containing protease inhibitors. Antibodies against proteins, including rabbit anti-KPNA2 (#14,372, CST), rabbit anti-CHEK2 (13,954–1-AP, proteintech), rabbit anti-FOXM1 (#20,459, CST), were incubated with the lysates for immunoprecipitation. The eluates were subsequently analyzed by Western blotting with antibody [mouse anti-KPNA2 (10,819–1-AP, proteintech); mouse anti-CHEK2 (#3440, CST); rabbit anti-FOXM1 (#20,459, CST)].

## Results

### Expression of KPNA2 in lung adenocarcinoma

**Fig. 1:**
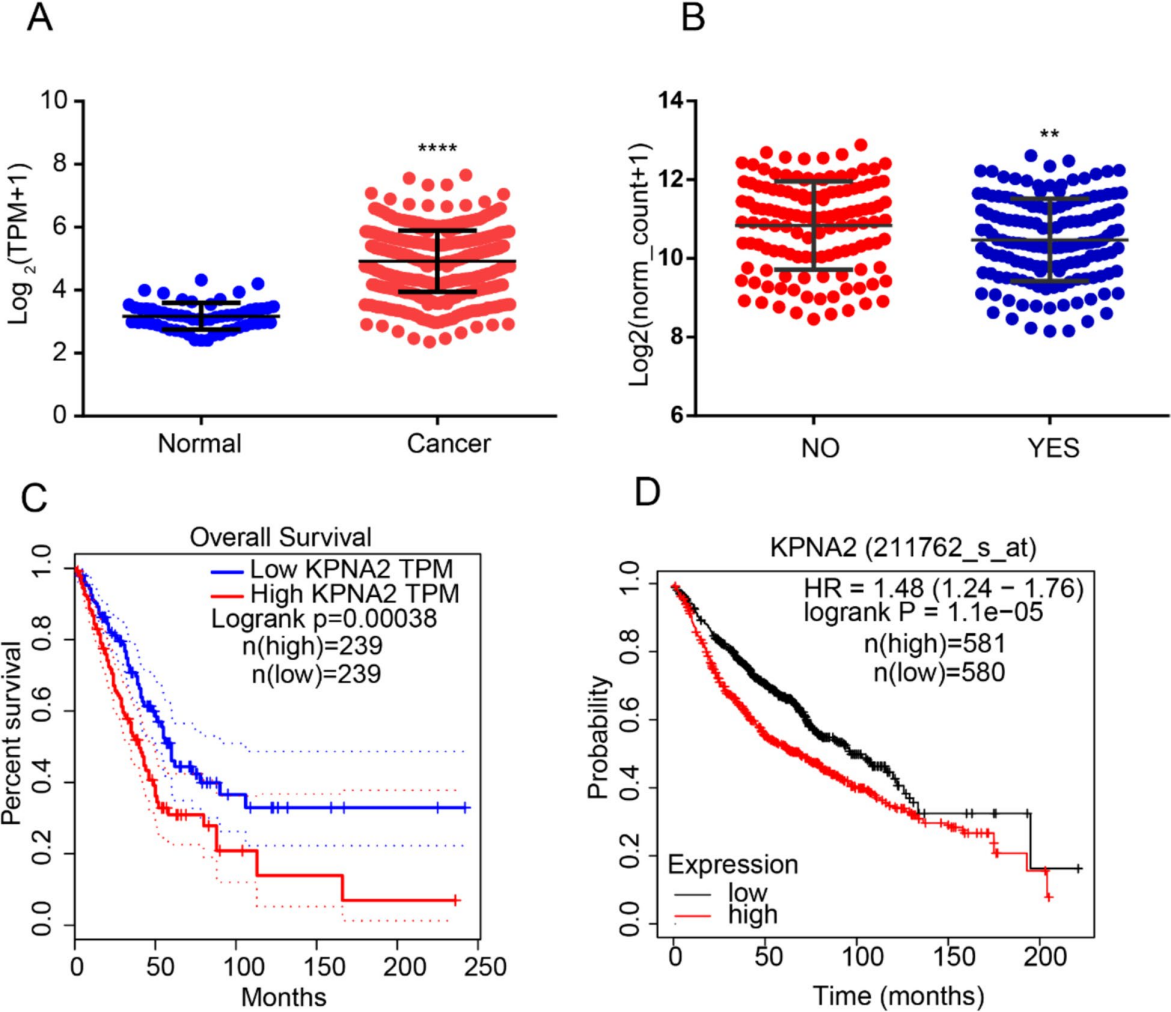
Expression of KPNA2 in lung adenocarcinoma. **A** KPNA2 is highly expressed in tumor tissues. **B** Cnncl_mt_n_KRAS_EGFR_ALK_RET_ROS1_BRAF_ERBB2_HRAS_NRAS_AKT1_MAP2 mutated group is lower than that of wild type. **C** Survival analysis using the GEPIA web. **D** Survival analysis using the kmplot web. **** *P* < 0.0001, ** *P* < 0.01 (mean ± SEM)

To investigate the effects of KPNA2 on lung adenocarcinoma, we downloaded data relating to lung adenocarcinoma from TCGA via UCSC Xena web and compared the expression of KPNA2 in normal and tumor tissues, on the basis of which, we established that KPNA2 is highly expressed in tumor tissues (Fig. 1A). Further analysis revealed that the expression in Cnncl_mt_n_KRAS_EGFR_ALK_RET_ROS1_BRAF_ERBB2_HRAS_NRAS_AKT1_MAP2 mutated cells was lower than that in wild-type cells (Fig. 1B). Analysis using GEPIA web revealed that patients with high expression of KPNA2 typically have a poor prognosis (Fig. 1C). Similarly, survival analysis using http://kmplot.com/analysis/index.php revealed that patients with a high expression of KPNA2 have a poor prognosis (Fig. 1D).

### KPNA2 promotes proliferation of lung adenocarcinoma

**Fig. 2:**
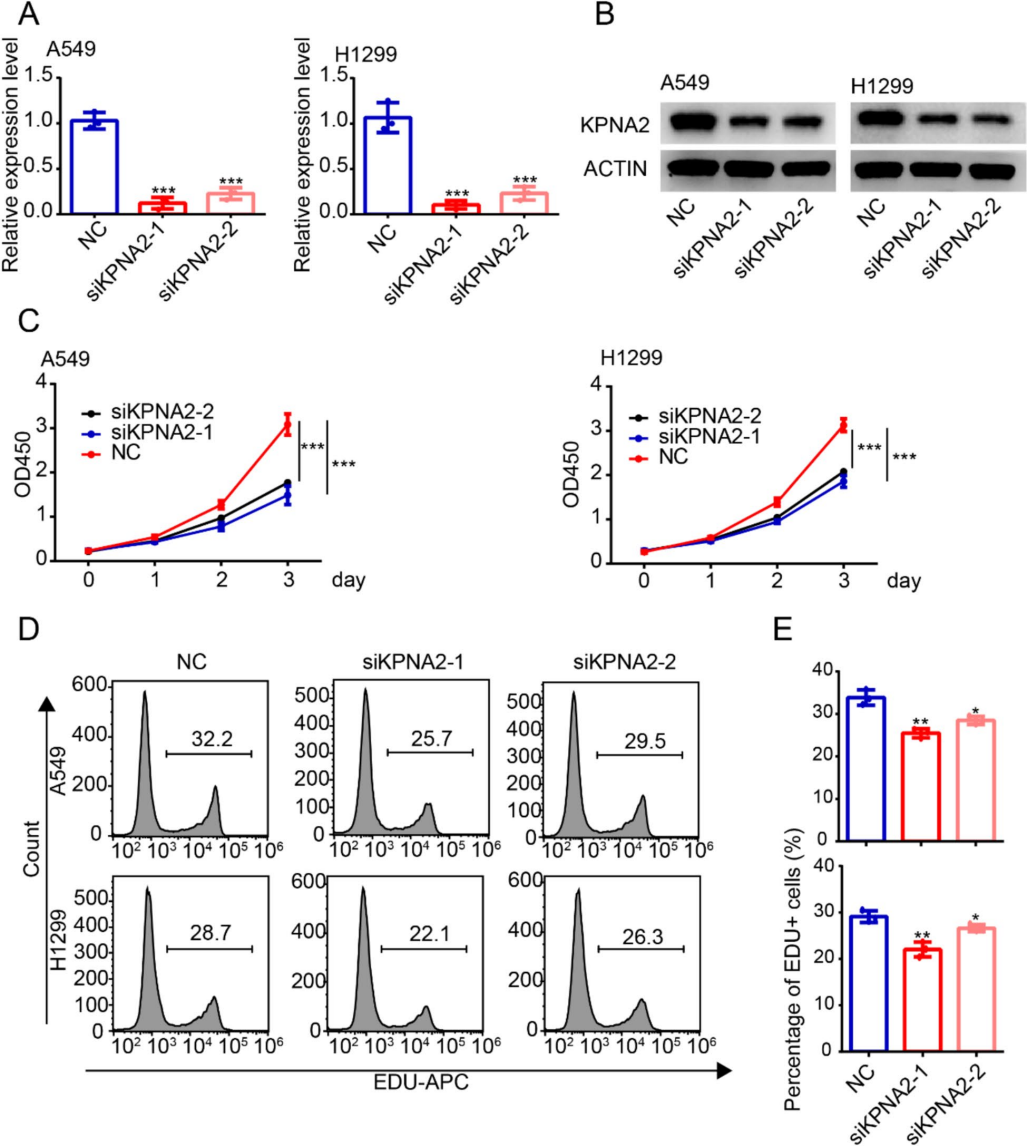
KPNA2 promotes the proliferation of lung adenocarcinoma. **A**, **B** qRT–PCR (**A**) and western blot (**B**) analyses of KPNA2 in A549 and H1299 cells transfected with control siRNA or siKPNA2. **C, D** Rates of cell proliferation determined using CCK-8 (**C**) and EDU (**D**) assays. Experiments were repeated at least three times. *** *P* < 0.001, ** *P* < 0.01, * *P* < 0.05 (mean ± SEM)

To establish whether KPNA2 influences the proliferation of lung adenocarcinoma, cells we knocked down the expression of this protein in A549 and H1299 cells using two different siRNAs and sought to verify the efficiency of silencing by performing qRT–PCR and western blot analyses, The results of qRT–PCR (Fig. 2A) and western blotting (Fig. 2B) accordingly revealed a significant suppression of KPNA2 expression. Furthermore, the results of CCK-8 assays indicated a significant inhibition of the proliferation of A549 and H1299 cells following KPNA2 knockdown (Fig. 2C), whereas EDU analysis revealed reductions in numbers of positive cells among the A549 and H1299 cells, similarly indicating an inhibition of cell proliferation (Fig. 2D, E).

### Bioinformatics reveals a significant correlation between FOXM1 and KPNA2

**Fig. 3:**
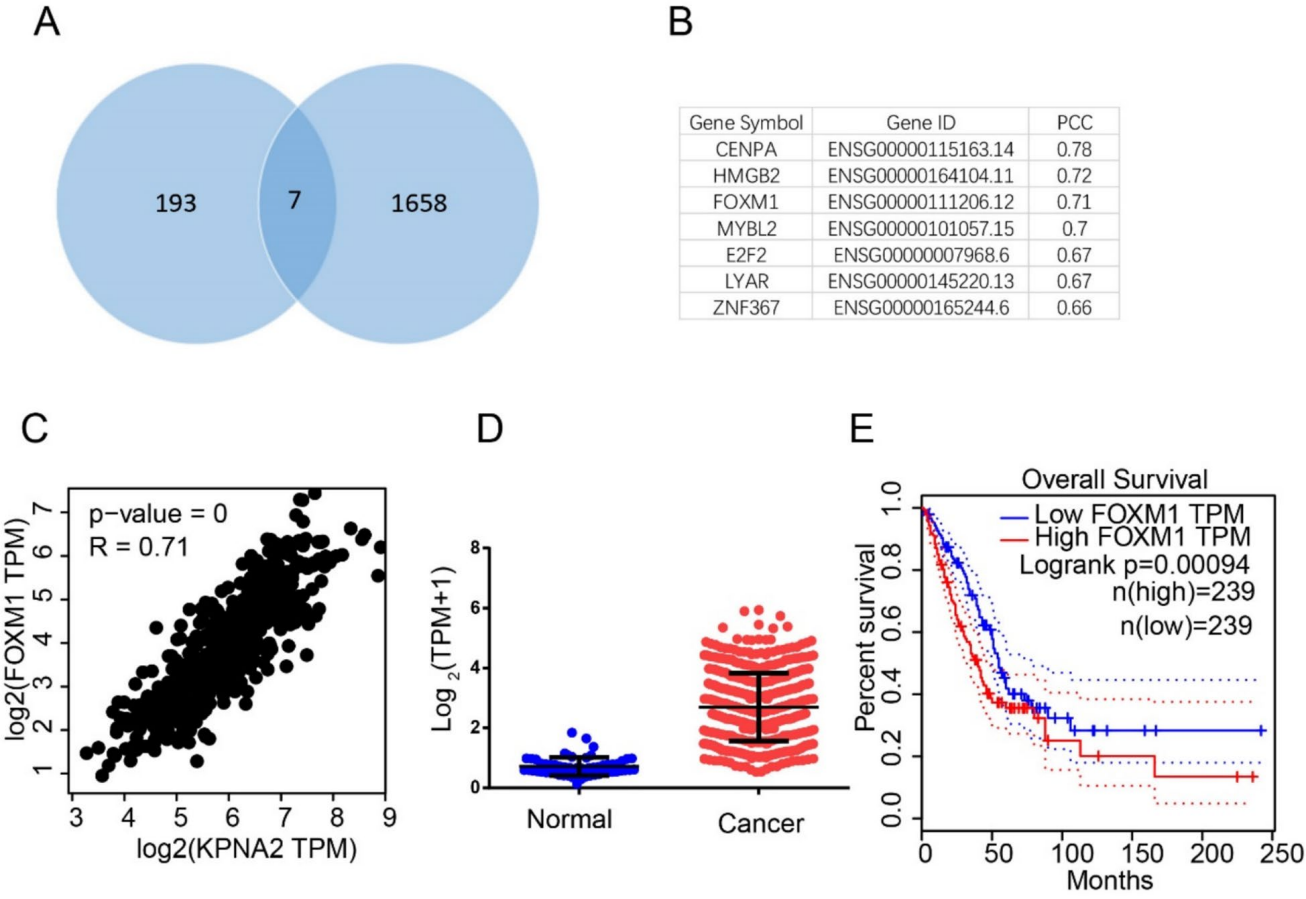
Bioinformatics analyses revealed a significant correlation between FOXM1 and KPNA2 expression. **A, B** On the basis of a screen of TCGA data, seven transcription factors were identified among the top 200 genes associated with KPNA2 expression in lung adenocarcinoma. **C** Correlation between FOXM1 and KPNA2. **D** Reference to the TCGA database indicated that compared with normal tissues, expression of FOXM1 was higher in lung adenocarcinoma. **E** Kaplan–Meier analysis of KPNA2 in the TCGA database

To elucidate the regulatory mechanisms of KPNA2 in lung adenocarcinoma, we further analyzed the top 200 genes associated with KPNA2 expression in lung adenocarcinoma obtained from the TCGA database, among which, we identified seven transcription factors (Fig. 3A). Of these, CENPA and HMGB2 are transcriptional cofactors that lack activation domains. Among the remaining factors, we selected FOXM1 for further analysis (Fig. 3C), with TCGA data indicating that FOXM1 is highly expressed in lung adenocarcinoma (Fig. 3D) and patients with a high expression have a poor prognosis (Fig. 3E).

### FOXM1 directly regulates the expression of KPNA2

**Fig. 4:**
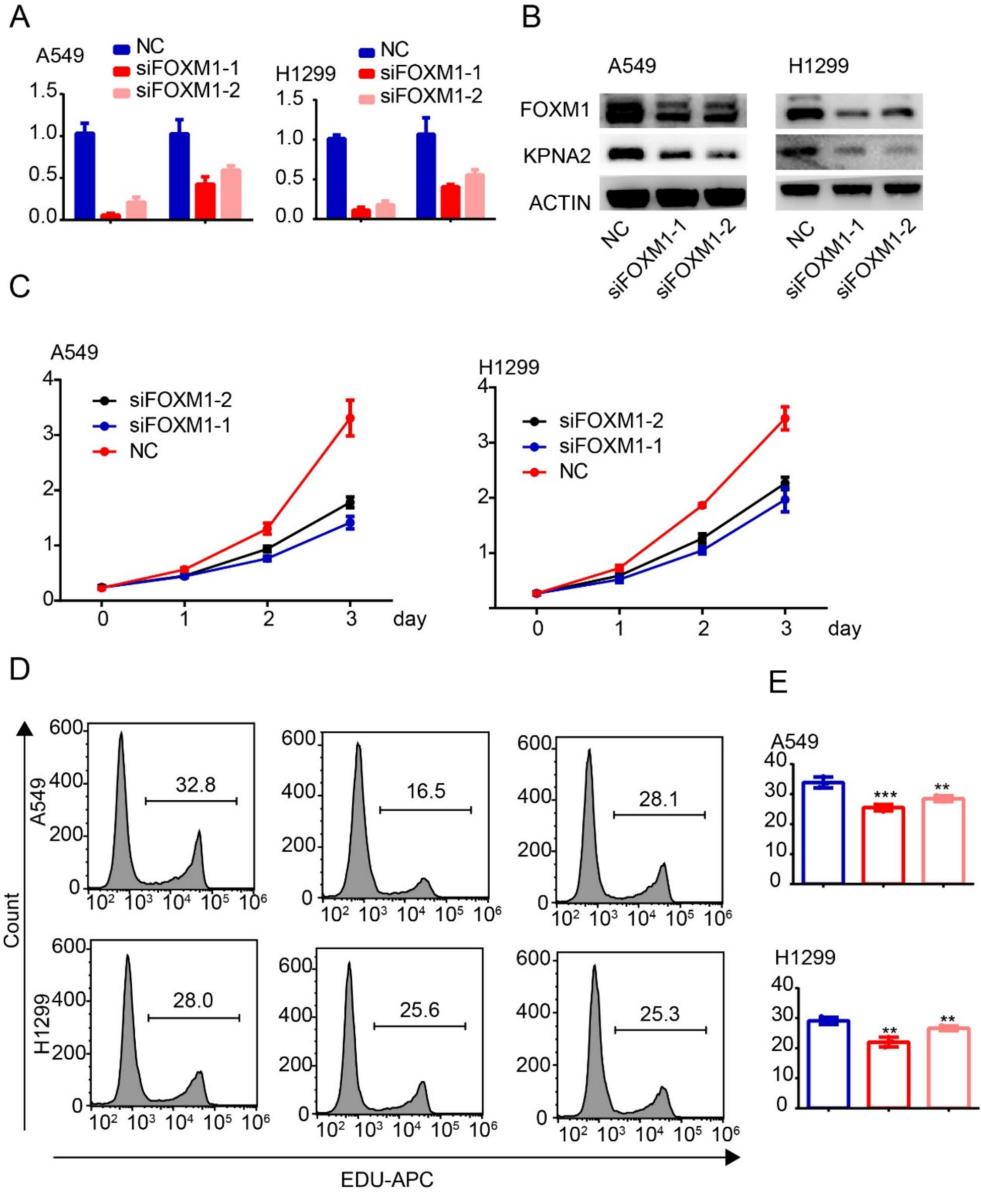
FOXM1 regulates the expression of KPNA2 and promotes proliferation of lung adenocarcinoma. **A, B** qRT–PCR (**A**) and western blot (**B**) analyses of FOXM1 in A549 and H1299 cells transfected with control siRNA or siFOXM1. **C, D** Rates of cell proliferation determined using CCK-8 (**C**) and EDU (**D**) assays. Experiments were repeated at least three times. *** *P* < 0.001, ** *P* < 0.01, * *P* < 0.05 (mean ± SEM)

Knock down of the expression of FOXM1 using interference RNA revealed that the expression of KPNA2 was reduced at both the mRNA (Fig. 4A) and protein (Fig. 4B) levels, and the results of CCK-8 assays indicated a significant inhibition of the proliferation of both the A549 and H1299 cell lines (Fig. 4C). Consistently, EDU analysis revealed reductions in the number of positive A549 and H1299 cells subsequent to FOXM1 knockdown, indicating an inhibition of cell proliferation (Fig. 4D, E).

### FOXM1 directly regulates the expression of KPNA2

**Fig. 5:**
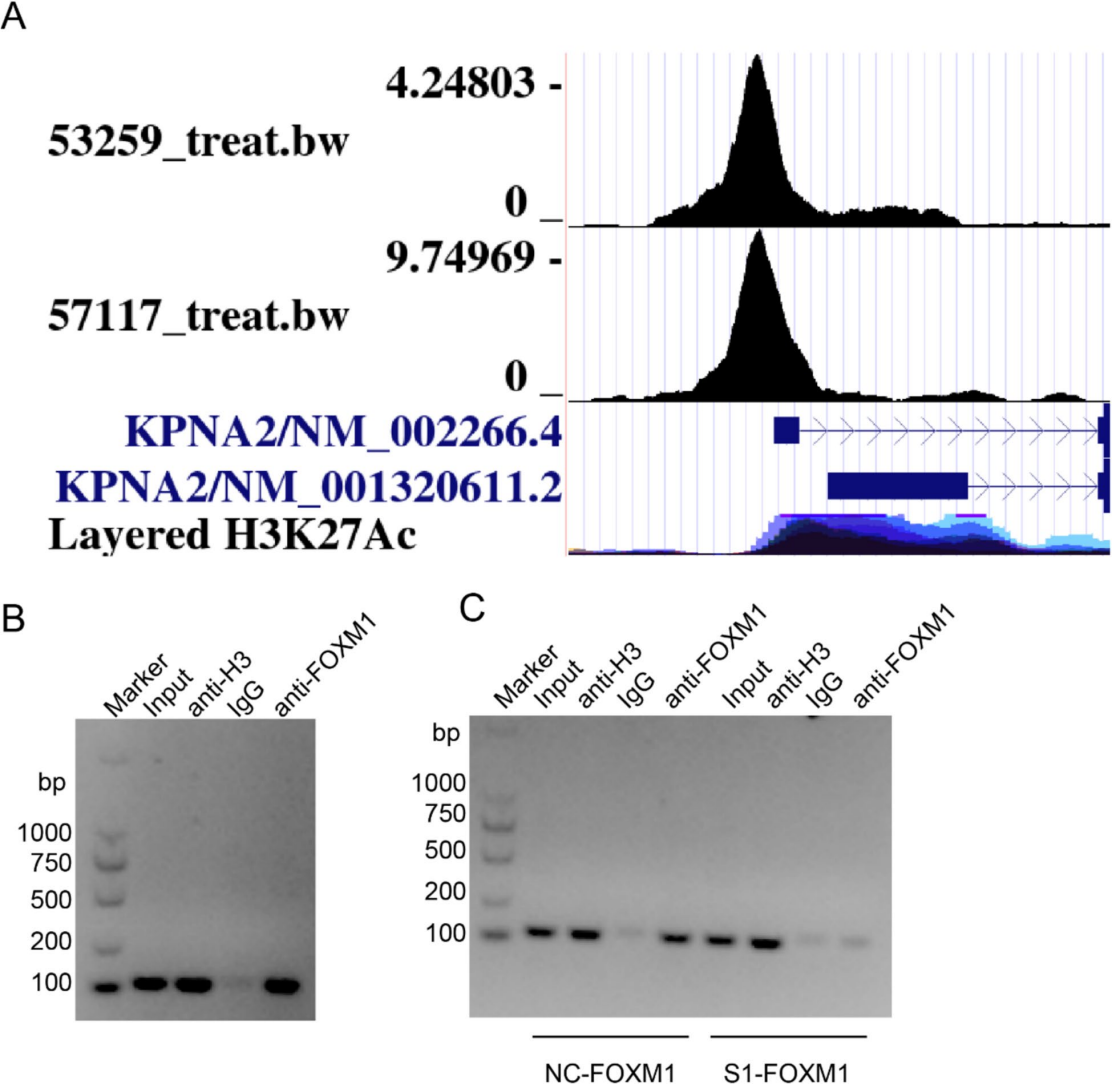
FOXM1 directly regulates the expression of KPNA2. **A** FOXM1 binding site in the KPNA2 promoter region. **B** Chromatin immunoprecipitation analysis of A549 cells. **C** Chromatin immunoprecipitation analysis of A549 cells transfected with control siRNA or siFOXM1-1

To further investigate the mechanisms underlying the regulation of KPNA2 mediated by FOXM1, we performed chromatin immunoprecipitation analysis using “HEK293; Epithelium; Embryonic Kidney” [13] and “MCF-7; Epithelium; Breast” [14] to assess whether FOXM1 can bind to the promoter sequence of KPNA2, with reference to the website http://cistrome.org/db/#/. This accordingly revealed that FOXM1 can bind to the promoter region of KPNA2 (Fig. 5A). Subsequently, we designed primers based on the binding sites and used an FOXM1 antibody for chromatin immunoprecipitation analysis using A549 cells, the findings of which consistently indicated that FOXM1 can bind to the promoter region of KPNA2 (Fig. 5B). This was further confirmed by our observation of a reduction in PCR products in cells following the knock down of FOXM1 (Fig. 5C). Collectively, these findings provide convincing evidence to indicate that the expression of KPNA2 is directly regulated by FOXM1.

### KPNA2 has a positive feedback effect on the expression of FOXM1

**Fig. 6:**
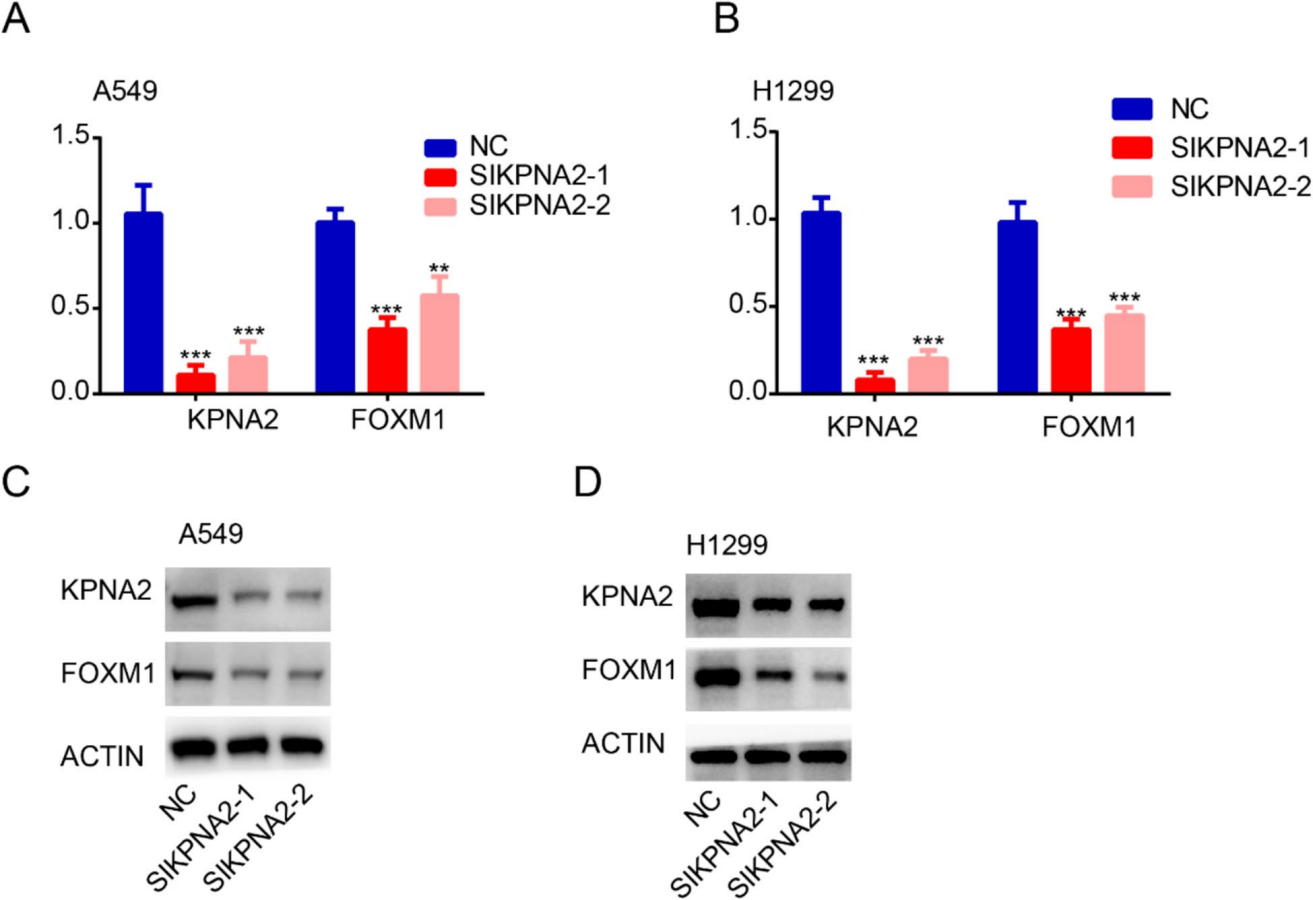
KPNA2 has a positive feedback effect on the expression of FOXM1. **A, B** qRT–PCR analysis of FOXM1 in A549 cells (**A**) and H1299 cells (**B**) transfected with control siRNA or siKPNA2. **C, D** Western blot analysis of FOXM1 in A549 cells (**C**) and H1299 cells (**D**) transfected with control siRNA or siKPNA2

**Fig. 7:**
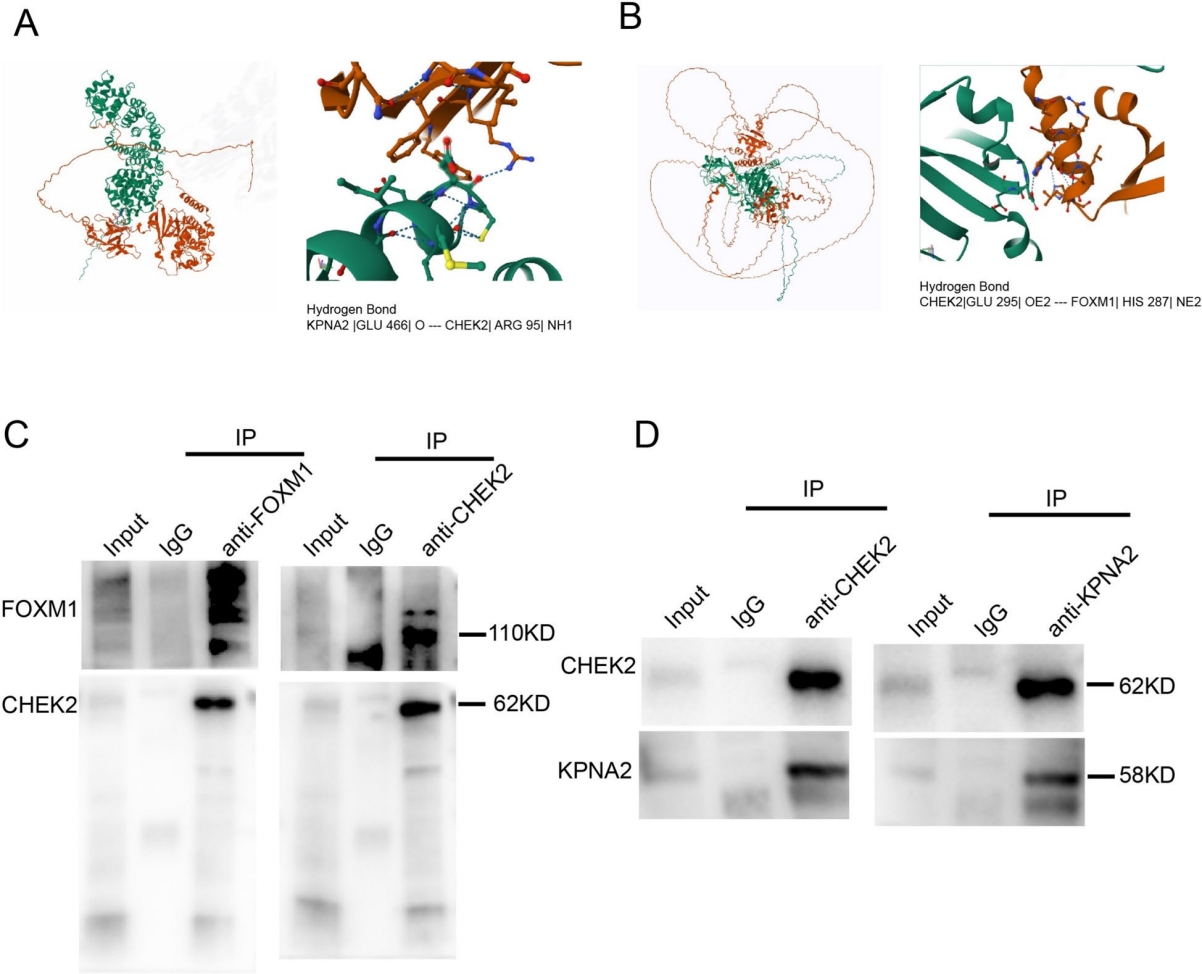
AlphaFold3 prediction of the binding sites of proteins in positive feedback interactions. **A, B** AlphaFold3 prediction of sites mediating the binding between KPNA2 and CHEK2 (**A**) and CHEK2 and FOXM1 (**B**)

We found that knocking down the expression of KNPA2 in the A549 and H1299 cell lines led to a significant reduction in FOXM1 expression at the mRNA level (Fig. 6A, B). Subsequent western blot analysis consistently confirmed a reduction in FOXM1 expression at the protein level in A549 and H1299 cell lines following the knock down of KPNA2 expression (Fig. 6C, D). Previously, it has also been established that KPNA2 can bind to CHEK2, promoting its entry into the nucleus [15], and that CHEK2 can in turn promote the stability of FOXM1 [16]. Moreover, it has been demonstrated that FOXM1 has a self-regulatory capacity. On the basis of these findings, we used AlphaFold3 to predict the sites associated with the binding between KPNA2 and CHEK2 and CHEK2 and FOXM1, which indicated that KPNA2 and CHEK2 can bind via the hydrogen bond KPNA2 |GLU 466| O–CHEK2| ARG 95| NH1 (Fig. 7A) and CHEK2 and FOXM1 can bind via the hydrogen bond CHEK2|GLU 295| OE2 –- FOXM1| HIS 287| NE2 (Fig. 7B). COIP assay showing the interaction between FOXM1 and CHEK2 (Fig. 7C). A549 cells were subjected to immunoprecipitation with rabbit anti-FOXM1 antibody or rabbit anti-CHEK2 antibody and anti-IgG antibody, followed by western blot analysis with mouse anti-CHEK2 antibody and rabbit anti-FOXM1 antibody. COIP assay showing the interaction between KPNA2 and CHEK2 (Fig. 7D). A549 cells were subjected to immunoprecipitation with rabbit anti-KPNA2 antibody or rabbit anti-CHEK2 antibody and anti-IgG antibody, followed by western blot analysis with mouse anti-CHEK2 antibody and mouse anti-KPNA2 antibody. These findings thus provide evidence that KPNA2 regulates the expression of FOXM1 via the CHEK2 pathway.

## Discussion

The precise regulation of signaling pathways is essential for maintaining cellular homeostasis, and the dysregulation of such pathways often leads to abnormal cell states. Cell signal transduction is regulated via two main feedback mechanisms, namely, negative and positive feedback. Negative feedback, as a key regulatory mechanism, is used to inhibit abnormal cell proliferation. For example, in response to growth factor stimulation, activation of the MAPK–ERK1/2 pathway will lead to a high expression of EGR1, thereby promoting rapid cell proliferation. In turn, however, an increase in EGR1 levels induces the upregulated expression of NAB1 and NAB2, which bind to the promoter of EGR1 and inhibit its transcription, thereby precisely regulating the rate of cell proliferation [17]. In contrast, positive feedback often leads to excessive cell proliferation, a prominent example of which is tumor formation, as exemplified by the VHL–histone lactylation–PDGFRβ positive feedback loop that contributes to accelerating the progression of clear cell renal cell carcinoma [18]. Similarly, the CENPU/E2F6/E2F1 positive feedback loop promotes proliferation and metastasis via the ubiquitination of E2F6 in HCC [19] and that of FAM83A/PI3K/AKT/c-Jun promotes migration, invasion, and metastasis in HCC [20]. Moreover, it has been found that the nuclear accumulation of KPNA2 influences radio-resistance through positive regulation of the PLSCR1–STAT1 loop in lung adenocarcinoma [21], whereas the IMPDH1/YB-1 positive feedback loop is associated with the assembly of cyto-ophidia and is considered a promising therapeutic target in metastatic tumors [22]. Further examples include a hypoxia-induced HIF1α-circTDRD3 positive loop that exacerbates colorectal cancer growth and metastasis [23], and a positive feedback loop between EBP2 and c-Myc that contributes to regulating rDNA transcription, cell proliferation, and tumorigenesis [24], whereas comprehensively mapped feedback loops between LncRNA PTENP1 and miR-21/PTEN have been implicated in the regulation of epithelial–mesenchymal transition and drug resistance in NSCLC [25]. Collectively, these examples provide a valuable context for placing the KPNA2/FOXM1 feedback loop within the broader landscape of self-reinforcing oncogenic networks.

Our research has revealed that KPNA2 is significantly overexpressed in lung adenocarcinoma tissues, and that patients with elevated levels of KPNA2 expression typically have poorer survival outcomes, which is consistent with the findings of previous studies. In NSCLC, a downregulation of KPNA2 leads to the downregulation of Oct4, thereby inhibiting cell proliferation [26], and miR-101 has been demonstrated to target KPNA2 to inhibit the progression of lung squamous cell carcinoma cell lines [27], whereas hsa_circ_0022383 promotes NSCLC tumorigenesis by regulating the miR-495–KPNA2 axis and the knockdown of KPNA2 has similarly been found to inhibits the proliferation and migration of lung cancer cells [28]. Moreover, in lung cancer cells, the knockdown of KPNA2 has been shown to be associated with a subcellular redistribution of E2F1. Quantitative proteomics analysis of the regulation of KPNA2 and its potential novel cargo proteins in NSCLC has revealed that the infiltration of macrophages is negatively correlated with patient prognosis in lung adenocarcinoma, and on the basis of cell cluster markers and genes associated with tumor-associated macrophages, it has been found that the expression patterns of *C1QTNF6*, *CCNB1*, *FSCN1*, *HMMR*, *KPNA2*, *PRC1*, *RRM2*, and *TK1* genes, as well as a risk scoring model, can all contribute to predicting the prognosis of the disease [29]. Notably, levels of KPNA2 protein in the pleural effusions obtained from patients with NSCLC have been found to be substantially higher compared with those from individuals without lung cancer, and knockdown of KPNA2 in lung cancer cells inhibits cell migration and reduces their viability [30]. These findings accordingly provide convincing evidence that KPNA2 plays a pivotal role in the development of lung adenocarcinoma and is controlled by a network of genes and micro- and circular RNAs.

Given that transcription factors that directly regulate the transcription of a particular gene generally show the strongest correlation with the expression of that gene, we sought to assess the direct regulation of KPNA2 using TCGA data, and accordingly identified CENPA, HMGB2, and FOXM1 as the top three transcription factors showing the highest consistency with KPNA2 expression. Among these, although CENPA and HMGB2 are able to bind to DNA, they lack transcriptional activation capacity, whereas FOXM1 contains both DNA binding and activation domains and can directly regulate gene expression, thereby identifying this factor as the primary candidate for a direct regulator of KPNA2. Moreover, our findings are consistent the operation of a positive feedback loop between the expression of KPNA2 and FOXM1, which contributes to enhancing the proliferation of lung adenocarcinoma cells. In the future, we intend to focus on further elucidating the molecular mechanism underlying the KPNA2–FOXM1 axis. Specifically, we will employ site-directed mutagenesis coupled with co-immunoprecipitation to map the precise sites of the binding between KPNA2 and CHEK2, and between CHEK2 and FOXM1. In addition, we hope to validate the role of CHEK2 within this feedback loop based on knockdown and subsequent rescue experiments in conjunction with the overexpression CHEK2.

FOXM1, which is upregulated in multiple tumors, plays a key role in promoting cancer cell proliferation. We found that knockdown of FOXM1 was associated with a significant reduction in the levels of KPNA2 expression, and the results of bioinformatics analysis performed using the GEPIA web indicated that FOXM1 may bind to the promoter region of KPNA2, thus providing evidence that this transcription factor might directly regulate the expression of KPNA2. To verify this conjecture, we designed specific primers and confirmed that FOXM1 does indeed bind to the KPNA2 promoter. Given our previous findings of a significant reduction in the expression of FOXM1 following the knock down of KPNA2, we speculate that KPNA2 and FOXM1 are mutually regulated via a positive feedback loop. In this regard, the findings of previous research indicate that KPNA2 interacts with CHEK2 to promote its nuclear translocation [15], whereas CHEK2 stabilizes FOXM1, thereby activating DNA repair-related genes [31]. Furthermore, the miR-577/CHEK2/FOXM1 axis has been linked to radiation resistance in clear cell renal cell carcinoma [32].

We believe that our findings in this study may have clinical relevance with respect to a number of key aspects. From the perspective of the direct targeting of molecules, it has been demonstrated that the inhibition KPNA2 (e.g., via the PRDM1/c-FOS pathway) has antitumor effects in bladder cancer models [33]. In addition, it has been established that the E3 ligase TRIM21 can disrupt the ABHD11–AS1/FOXM1 feedback loop by degrading the FOXM1-stabilizing factor IGF2BP2, thereby providing a novel intervention target [34]. With respect to combination therapy, in breast cancer, it has been demonstrated that activation of the FOXM1–CDK1 positive feedback loop induces replication stress, and that targeting the MMB–FOXM1 complex (e.g., LIN54) contributes to enhancing the efficacy of CHK1 inhibitors [35]. It is thus conceivable that similar strategies might be applicable in the case of KPNA2/FOXM1-dependent tumors. Furthermore, in terms of epigenetic regulation, it has been found that the ASPM–FOXM1 dual positive feedback loop promotes the progression of liver cancer via liquid–liquid-phase separation mechanisms, and that targeting this mechanism (e.g., by disrupting phase separation) may represent a novel therapeutic strategy [36].

## Conclusion

Our findings in this study, consistent with those reported previously, indicate that KPNA2 interacts with CHEK2, facilitating its nuclear translocation. In turn, CHEK2 stabilizes the FOXM1 protein, the overexpression of which directly modulates the expression of KPNA2, thereby establishing a positive feedback loop. It is envisaged that this mechanism contributes to the regulation of downstream genes associated with the cell cycle and thus promotes the proliferation of lung adenocarcinoma cells. However, these findings provide only a preliminarily verification of the regulatory effects of KPNA2 on FOXM1 expression, and, accordingly, further in-depth research is needed to elucidate specific mechanisms.

## Supplementary Information

Supplementary Material 1.

Supplementary Material 2.

Supplementary Material 3.

Supplementary Material 4.

Supplementary Material 5.

Supplementary Material 6.

Supplementary Material 7.

Supplementary Material 8.

Supplementary Material 9.

Supplementary Material 10.

Supplementary Material 11.

Supplementary Material 12.

Supplementary Material 13.

Supplementary Material 14.

## Data Availability

No datasets were generated or analysed during the current study.
