# Supplementary figures and images for: A positive feedback loop between KPNA2 and FOXM1 promotes the proliferation of lung adenocarcinoma
Source: Eur J Med Res. 2025 Dec 18;31:122. doi: 10.1186/s40001-025-03674-1 (PMC12829203; doi:10.1186/s40001-025-03674-1)

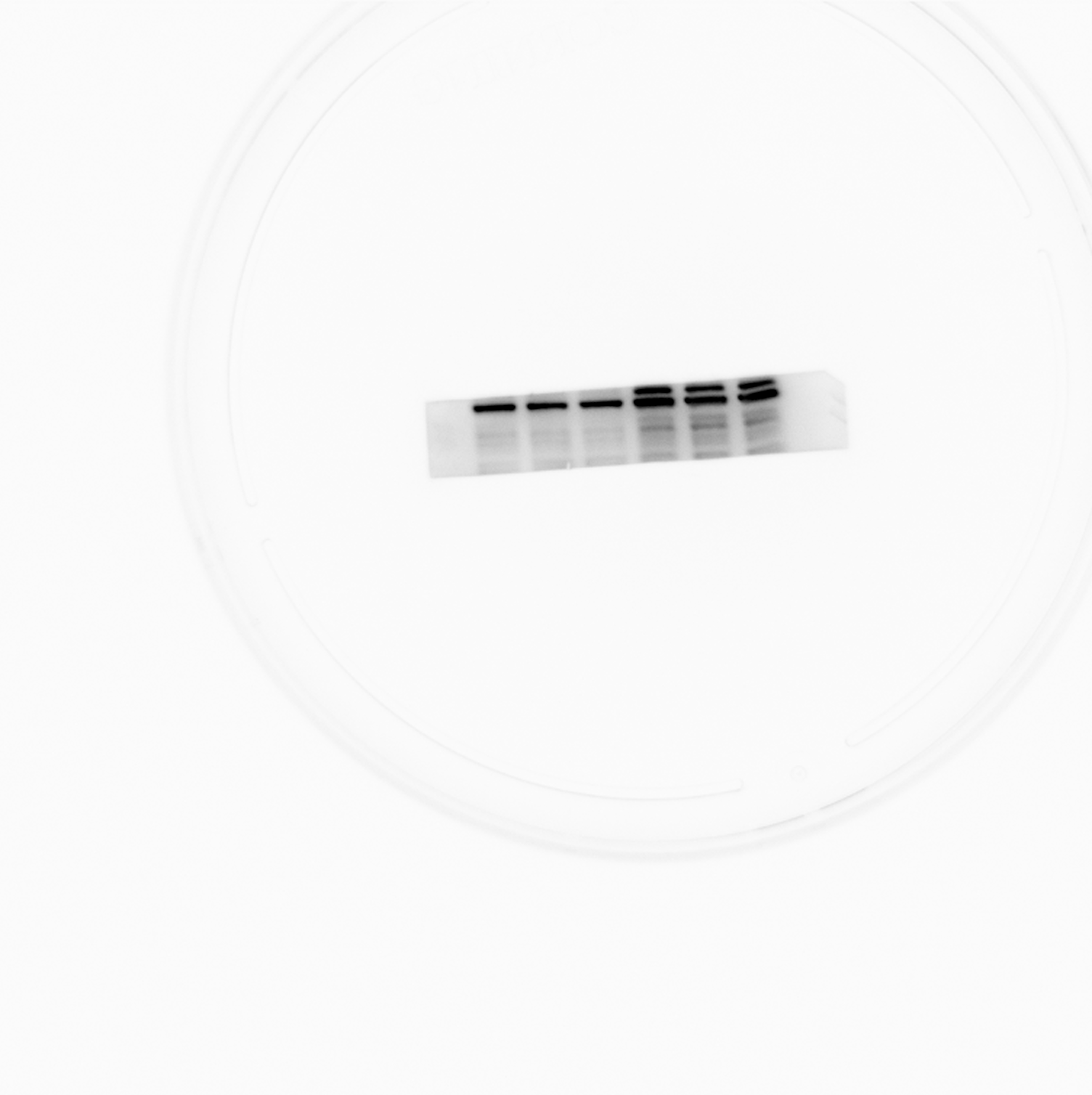

Supplement: Supplementary file 1 — Supplementary Material 1. [file 40001_2025_3674_MOESM1_ESM.tif]

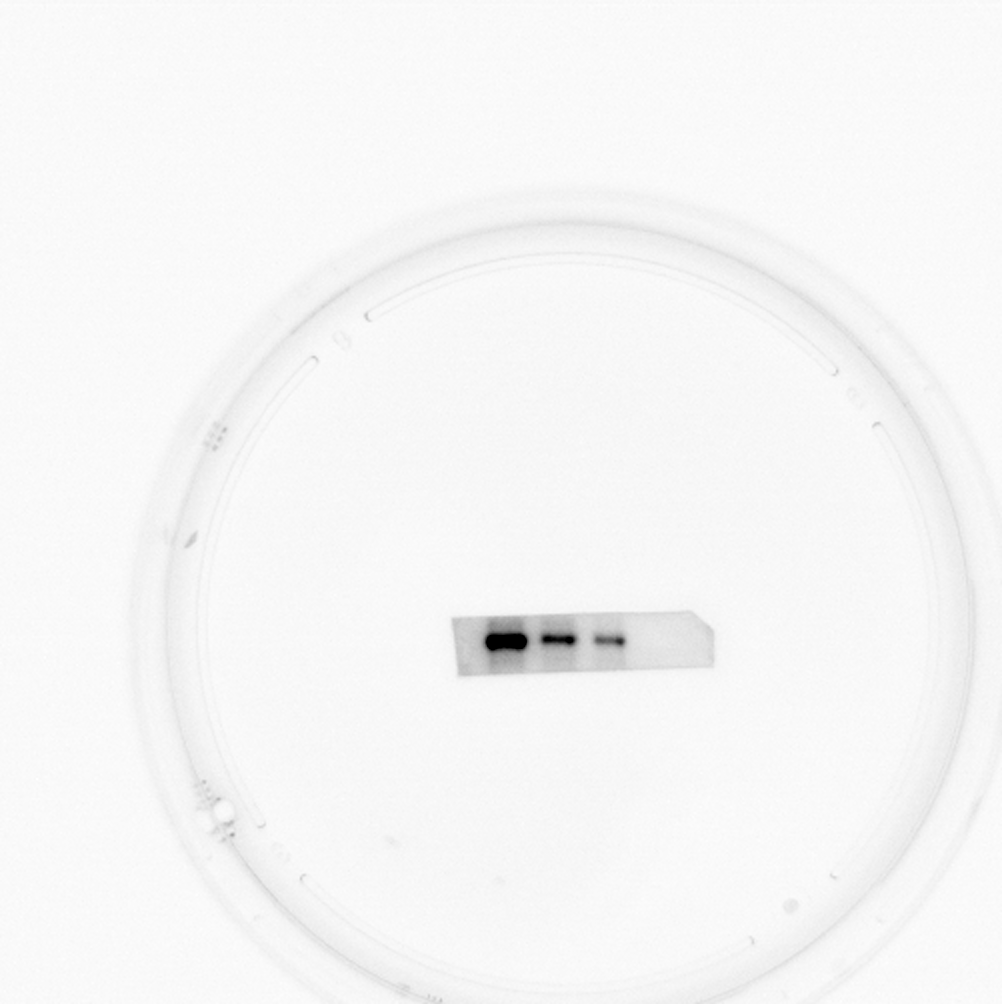

Supplement: Supplementary file 2 — Supplementary Material 2. [file 40001_2025_3674_MOESM2_ESM.tif]

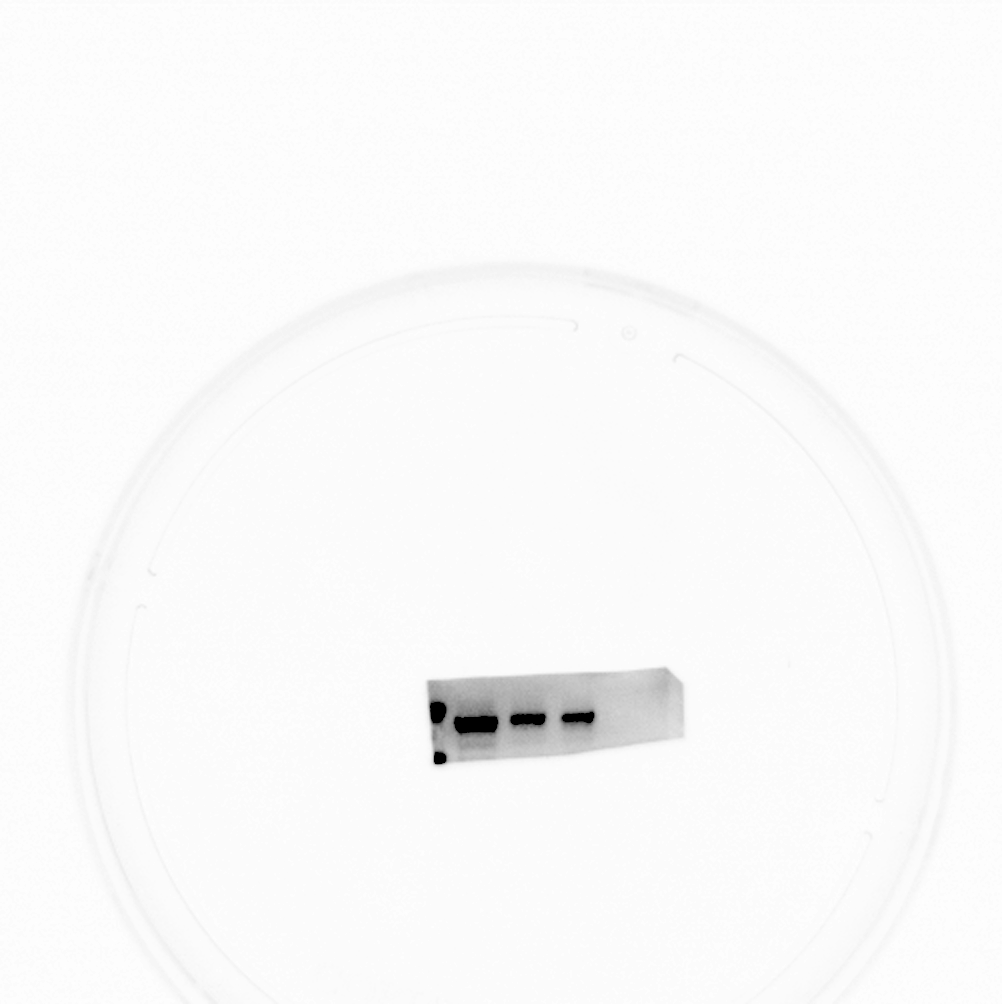

Supplement: Supplementary file 3 — Supplementary Material 3. [file 40001_2025_3674_MOESM3_ESM.tif]

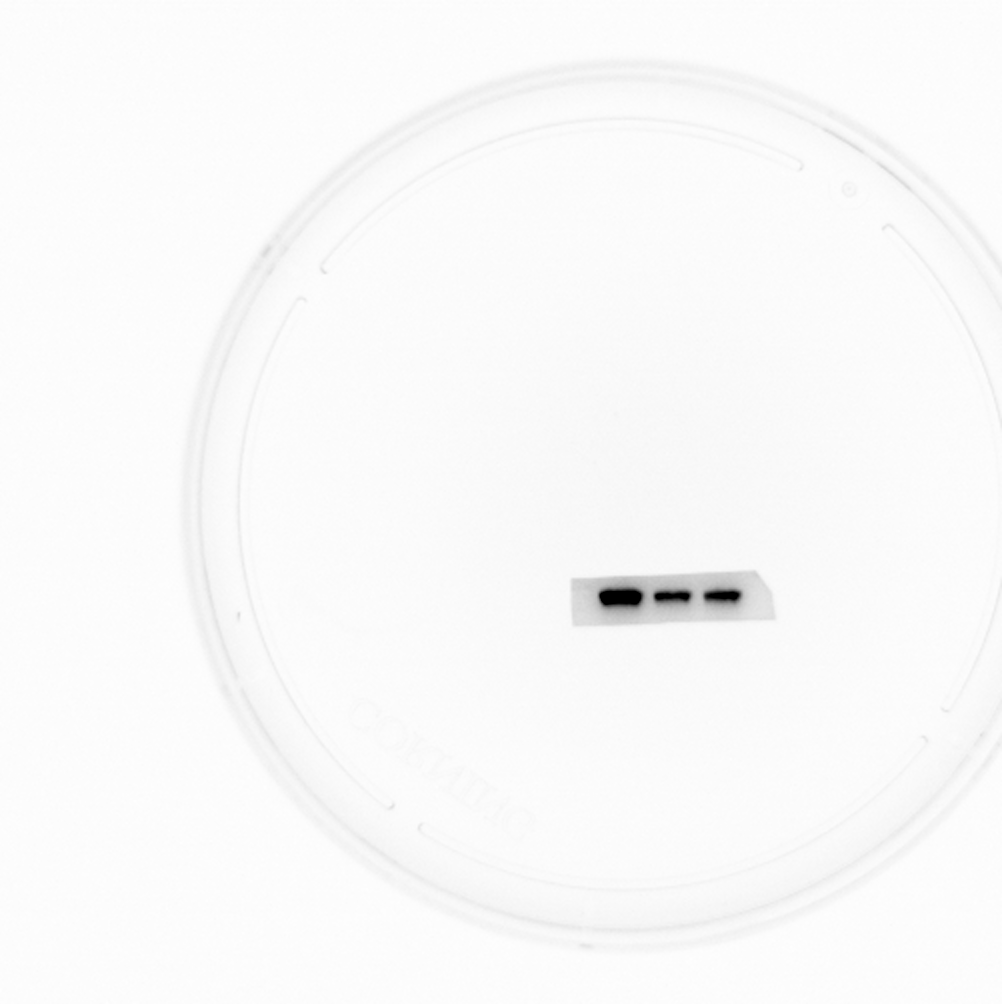

Supplement: Supplementary file 4 — Supplementary Material 4. [file 40001_2025_3674_MOESM4_ESM.tif]

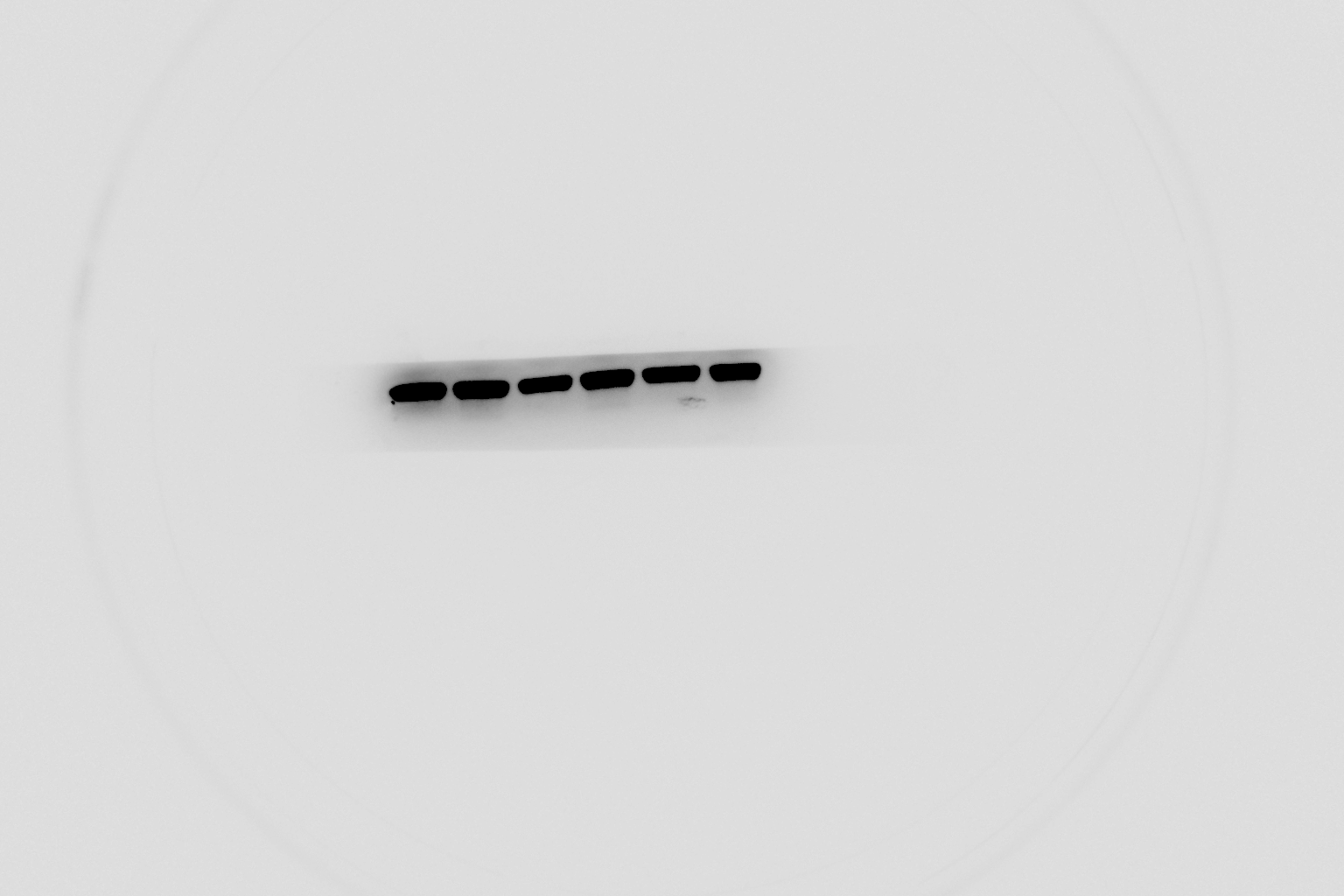

Supplement: Supplementary file 5 — Supplementary Material 5. [file 40001_2025_3674_MOESM5_ESM.tif]

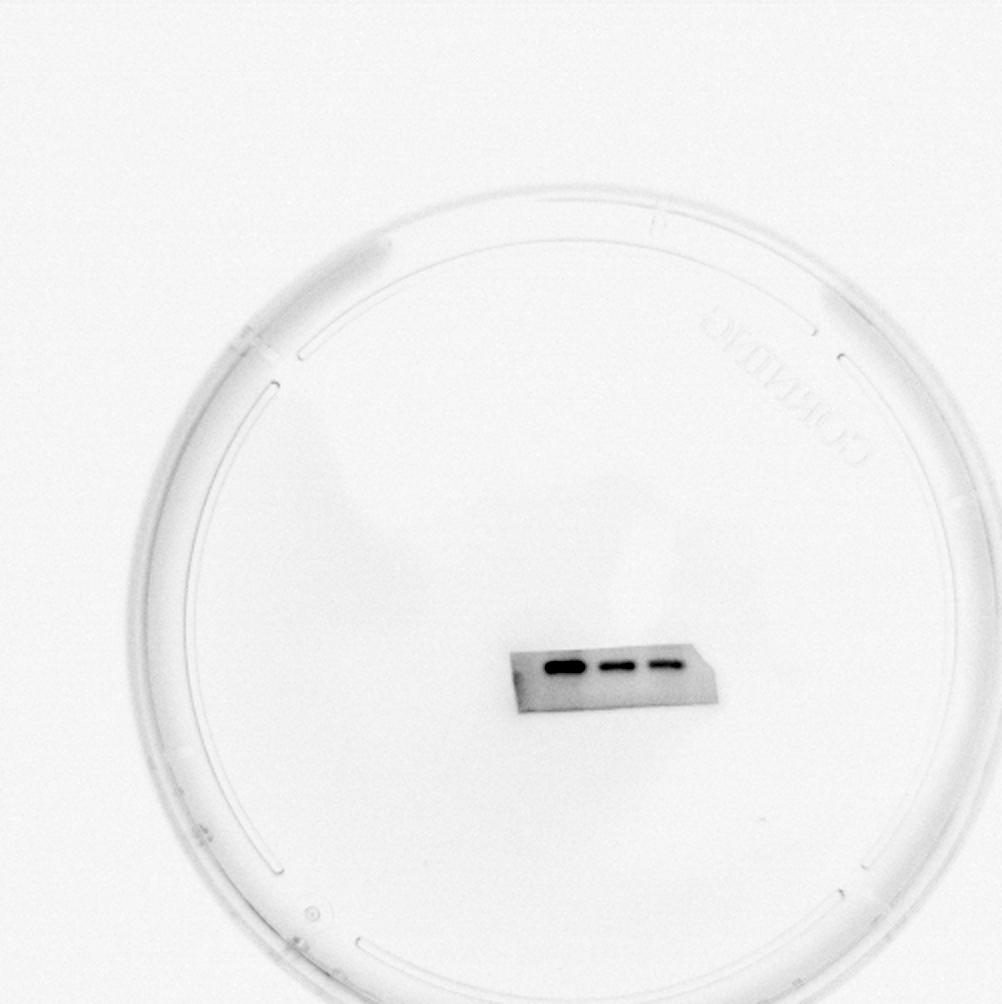

Supplement: Supplementary file 6 — Supplementary Material 6. [file 40001_2025_3674_MOESM6_ESM.tif]

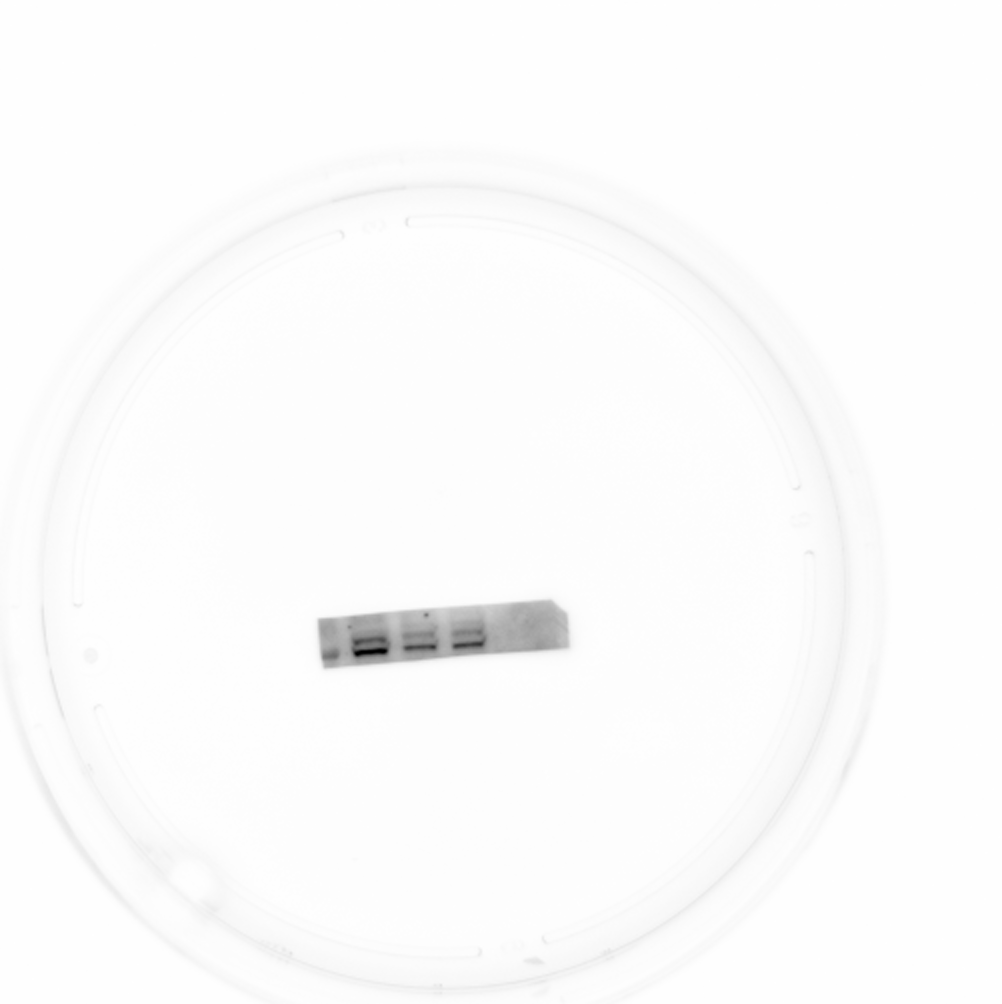

Supplement: Supplementary file 7 — Supplementary Material 7. [file 40001_2025_3674_MOESM7_ESM.tif]

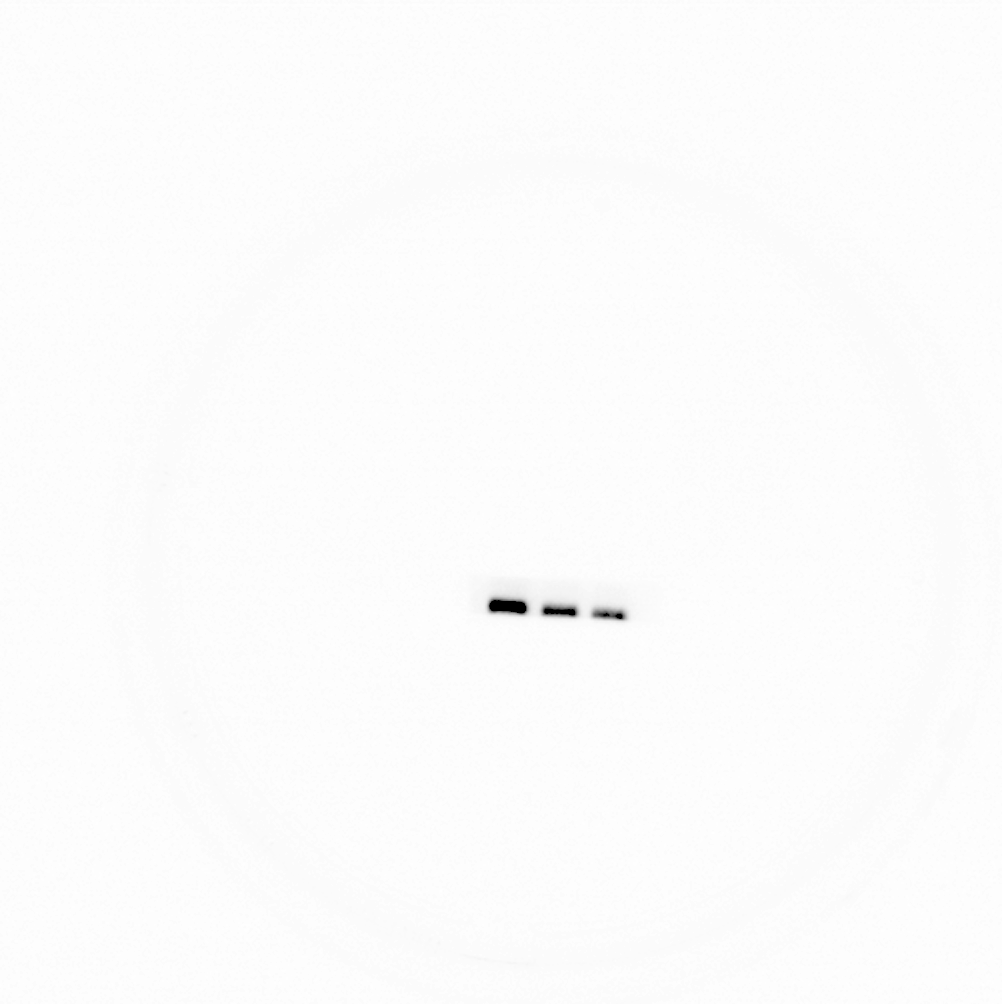

Supplement: Supplementary file 8 — Supplementary Material 8. [file 40001_2025_3674_MOESM8_ESM.tif]

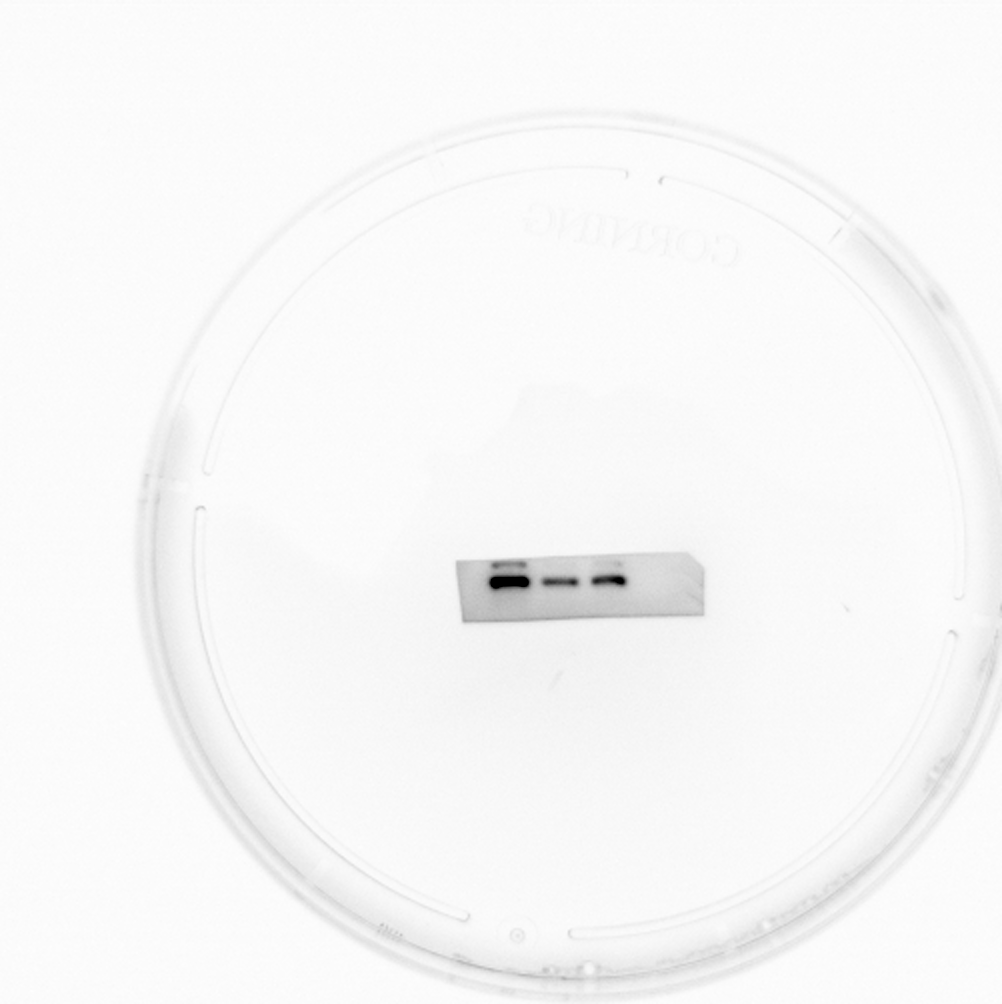

Supplement: Supplementary file 9 — Supplementary Material 9. [file 40001_2025_3674_MOESM9_ESM.tif]

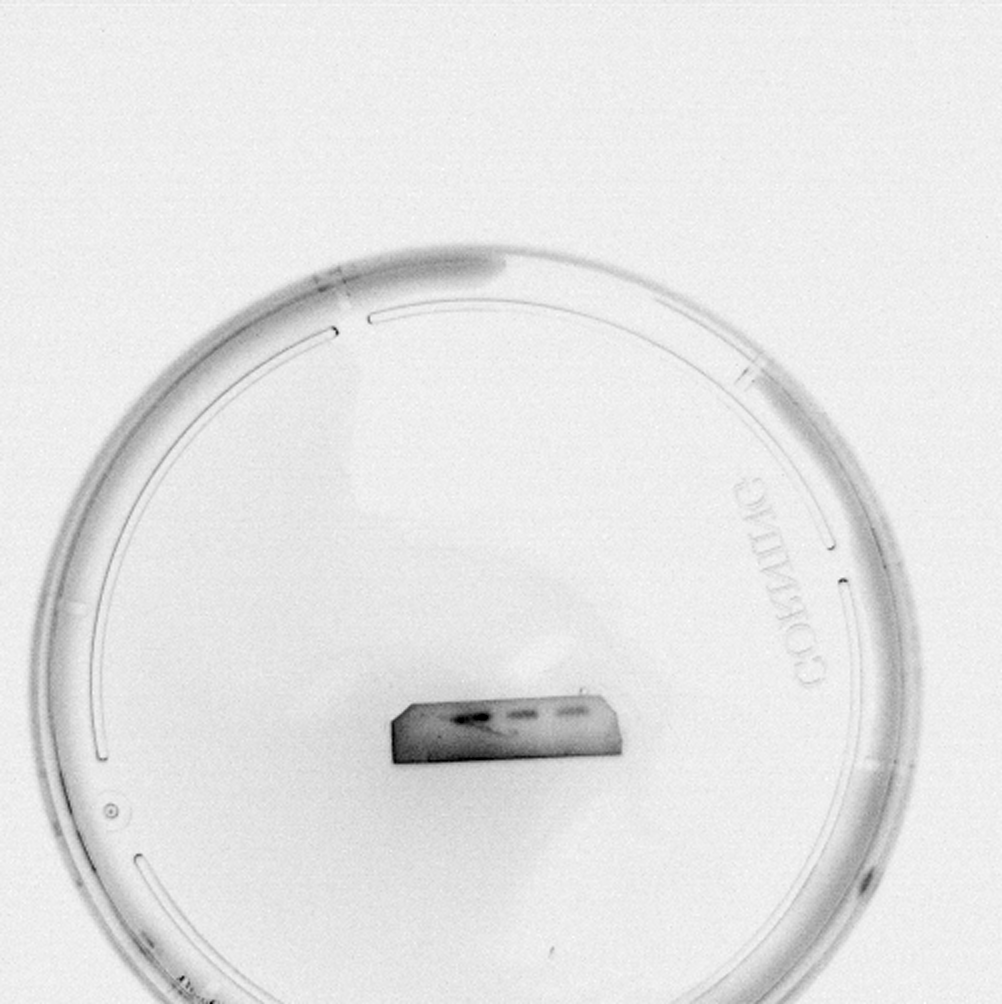

Supplement: Supplementary file 10 — Supplementary Material 10. [file 40001_2025_3674_MOESM10_ESM.tif]

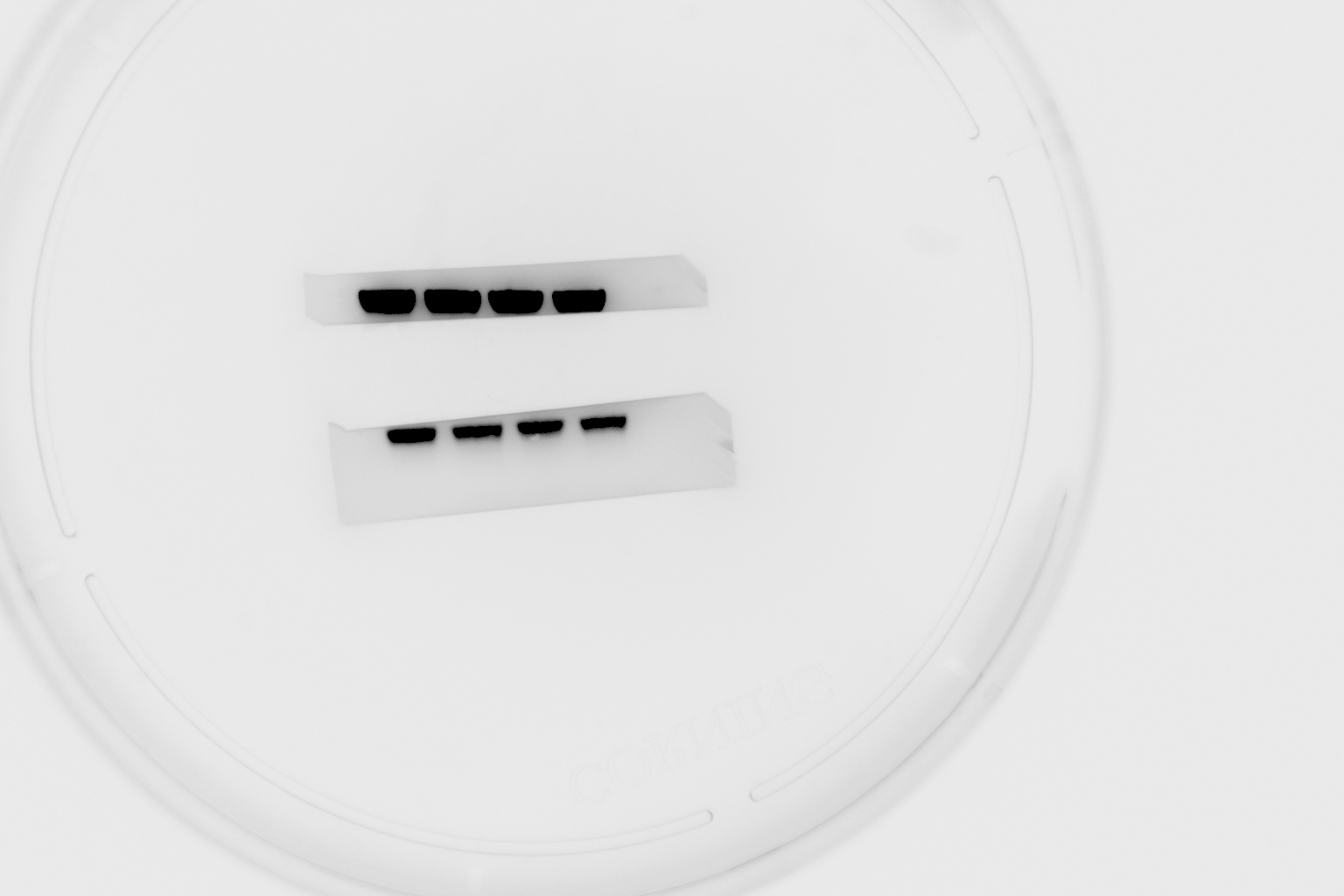

Supplement: Supplementary file 11 — Supplementary Material 11. [file 40001_2025_3674_MOESM11_ESM.tif]

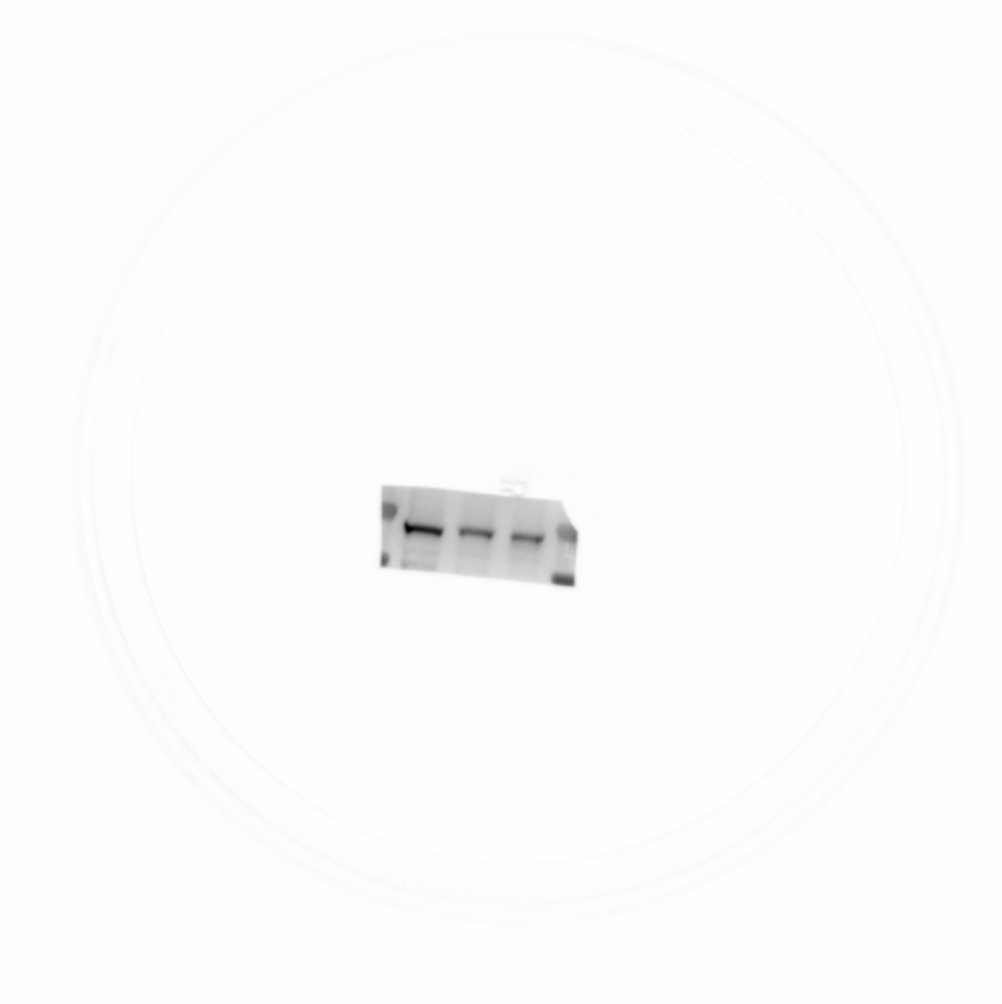

Supplement: Supplementary file 12 — Supplementary Material 12. [file 40001_2025_3674_MOESM12_ESM.tif]

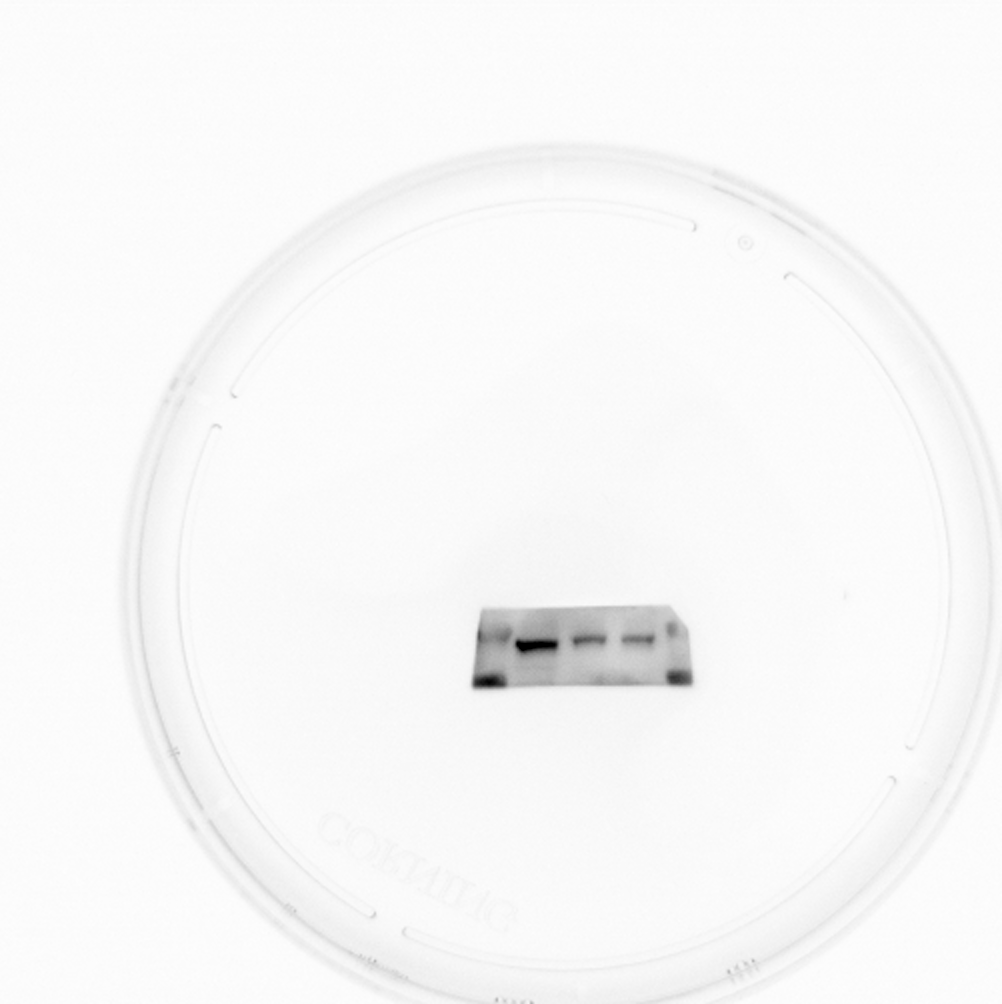

Supplement: Supplementary file 13 — Supplementary Material 13. [file 40001_2025_3674_MOESM13_ESM.tif]

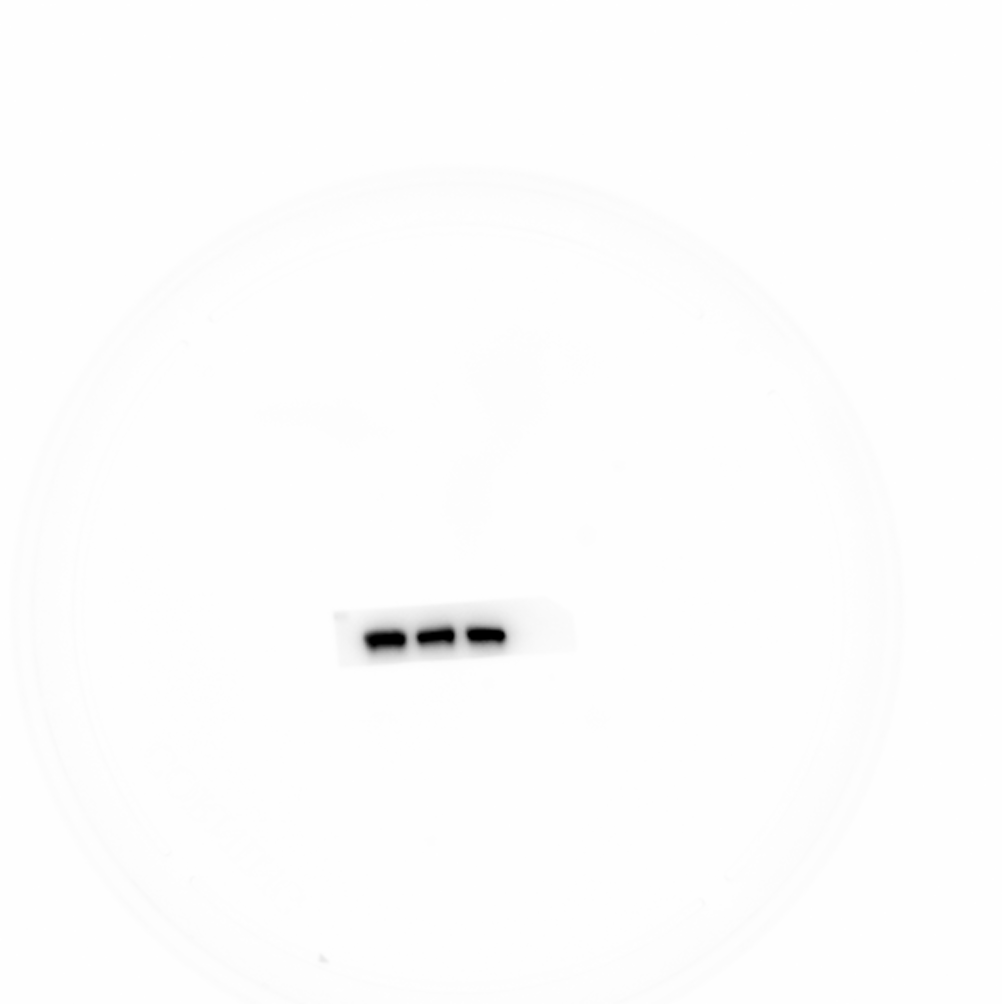

Supplement: Supplementary file 14 — Supplementary Material 14. [file 40001_2025_3674_MOESM14_ESM.tif]
